# Microbiota as Potential Functional Traits Facilitating Springtail Activity in Winter

**DOI:** 10.1002/ece3.71448

**Published:** 2025-05-19

**Authors:** Cao Hao, Bing Zhang, Pingting Guan, Zhijing Xie, Guoliang Xu, Donghui Wu, Ting‐Wen Chen

**Affiliations:** ^1^ School of Geography and Remote Sensing Guangzhou University Guangzhou China; ^2^ State Environmental Protection Key Laboratory of Wetland Ecology and Vegetation Restoration, School of Environment Northeast Normal University Changchun China; ^3^ Key Laboratory of Wetland Ecology and Environment, Northeast Institute of Geography and Agroecology Chinese Academy of Sciences Changchun China; ^4^ China Grassland Research Center, School of Grassland Science Beijing Forestry University Beijing China; ^5^ J.F. Blumenbach Institute of Zoology and Anthropology University of Göttingen Göttingen Germany

**Keywords:** comparative method, functional trait, gut microbiome, soil microarthropod, winter biodiversity

## Abstract

Understanding the role of microbiota in supporting animal survival and activity under extreme environmental conditions provides valuable insights into adaptation and resilience mechanisms in ecosystems. While vertebrate microbiota have received considerable attention, those associated with arthropods, particularly species capable of surviving sub‐zero temperatures, remain poorly understood. Springtails (Collembola), key contributors to litter decomposition and soil ecosystem functioning, require specialized adaptations to endure harsh winter conditions. Using the α‐ and β‐niche trait concept and phylogenetic comparative approaches, we investigated the microbiota of 10 coexisting springtail species with different overwintering strategies. Our results revealed that certain bacterial genera, including *Marmoricola*, *Mycobacterium*, *Rhodococcus*, and *Vibrionimonas*, exhibited phylogenetic signal, suggesting evolutionary constraints on their potential roles in hosts. Winter‐active springtail species harbored higher bacterial diversity and distinct microbial community compositions compared to inactive species, with enrichment in bacteria such as *Wolbachia*, *Morganellaceae*, and *Micrococcaceae*. Additionally, winter‐active species exhibited higher energy metabolism and lower lipid metabolism, alongside more frequent positive interactions within bacterial networks. These findings suggest that microbiota may play a functional role in supporting the metabolic demands of winter‐active springtails, potentially contributing to their adaptation to cold environments. Overall, our study highlights the role of microbiota in shaping ecological success and adaptation of arthropods to extreme conditions, providing new perspectives for soil animal research by integrating microbial functional traits with the evolutionary context of microbe‐host interactions.

## Graphical abstract

Microbiota in 10 springtail species were analyzed using phylogenetic comparative methods. Winter‐active species showed higher bacterial diversity, enriched with *Wolbachia*, *Morganellaceae*, and *Micrococcaceae*, while some bacteria exhibited a phylogenetic signal, suggesting evolutionary constraints. These microbiota may support host metabolism and adaptation to cold environments.

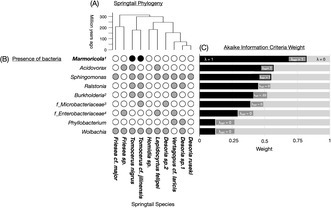

## 1 Introduction

The ability of animals to overwinter depends on adaptive changes in their genome, morphology, and physiology that enable survival under harsh winter conditions. These adaptations are crucial for maintaining community structure and ecosystem function in temperate and cold regions (Burns et al. 2010; Dietz et al. 2022). Some arthropods enhance their resistance to low temperatures by producing antifreeze proteins (Dageri et al. 2023; Scholl et al. 2023). Beyond genetic and physiological adaptations, animals also rely on symbiotic relationships with microbiota, which provide essential support for metabolism, immune defense, and stress tolerance (Ferguson et al. 2018; Mushegian and Tougeron 2019; Morgan‐Richards et al. 2023; Arango et al. 2024). In mammals, gut microbiota have been shown to enhance nutrient acquisition and energy homeostasis under cold conditions (Chevalier et al. 2015; Sommer et al. 2016; Regan et al. 2022). However, whether poikilothermal animals like arthropods benefit from their microbiota in similar ways remains largely unexplored, despite the fact that many species are active during winter and utilize resources from snow microhabitats (Hågvar 2010; Hao et al. 2020, 2022).

Here, we consider microbiota as functional traits that support animals in harsh environments, contributing to their coexistence within ecological communities. Microbiota assist hosts in degrading complex substrates and regulating physiological functions, ultimately benefiting host health and fitness. These microbial traits are shaped by multiple factors, including host phylogeny, feeding habits, ecological niches, and environmental conditions (Engel and Moran 2013; Gong et al. 2018; Hao et al. 2022). Across different host species, microbiota often exhibit phylogenetic signal, with closely related hosts tending to harbor similar microbial communities or share specific microbial taxa (Kudo et al. 2019). Such microbial taxa may have co‐evolved with their hosts over evolutionary timescales, resulting in stable associations. For example, Gong et al. (2018) demonstrated that bacterial differences between oribatid mite species are more strongly associated with host phylogeny than with trophic niche differences. In beetles, Kudo et al. (2019) found that the enrichment of the bacterial family *Enterobacteriaceae* was closely linked to host phylogeny.

Springtails (Collembola) are globally distributed soil microarthropods that play a key role in soil ecosystem processes, such as organic matter decomposition and nutrient cycling through microbial regulation (Bardgett and van der Putten 2014; Potapov et al. 2021). Some springtail species can tolerate sub‐zero temperatures and survive extreme winter conditions (Hao et al. 2020, 2024). Long‐term observations suggest that while some species enter dormancy or a chill coma state, others remain active during snow cover. Most studies on springtail‐associated microbiota have focused on the growing season (Bahrndorff et al. 2018; Zhu et al. 2021; Liu et al. 2024), with limited research on their role in host survival and adaptation during winter. A recent study on mosquitoes showed that differences in gut microbiota influence sugar reserves and lipid accumulation between diapausing and non‐diapausing individuals (Didion et al. 2021), suggesting that microbial contributions to host energy metabolism may be linked to life history strategies and phenology (Fu et al. 2020; Hao et al. 2024). For winter‐active springtails, distinct microbial communities may be essential to sustaining metabolic demands compared to their inactive counterparts.

In this study, we apply the α‐ and β‐niche trait framework to investigate phylogenetic signals in the microbiota associated with coexisting springtail species in winter. Functional traits, which define ecological niches shaped by past evolutionary processes, may exhibit varying degrees of phylogenetic signal. According to the α‐ and β‐niche trait concept, traits related to environmental tolerance (β‐niche traits) tend to show stronger phylogenetic signals than resource‐acquisition traits (α‐niche traits), which are partitioned among coexisting species to reduce competition (Silvertown et al. 2005; Chen et al. 2017; Noske et al. 2024). We tested whether the presence of certain bacterial taxa exhibit phylogenetic signal, suggesting that they function as β‐niche traits, which potentially influence host winter survival. To do so, we compared bacterial communities between five winter‐active springtail species (*Tomocerus* cf. *jilinensis*, *Desoria ruseki*, *Desoria* sp.1, *Desoria* sp.2 and *Vertagopus* cf. *laricis*) and five winter‐inactive species (*Tomocerus nigrus*, *Lepidocyrtus felipei*, *Homidia* sp., *Friesea* sp. and *Friesea* cf. *major*). We hypothesize that (1) some bacterial taxa exhibit phylogenetic signal, indicating co‐evolution with springtails, and (2) microbial diversity, community composition, network interaction, and potential metabolic functions differ between winter‐active and inactive species. By exploring these patterns, we aim to enhance our understanding of how microbiota contribute to animals adaptation in extreme environments and provide insights into their role in soil biodiversity and ecosystem functioning.

## 2 Materials and Methods

### 2.1 Study Site

We sampled springtails from the long‐term experimental station in Sanjiang Mire Wetland (47°35′ N, 133°31′ E), located in the center of the Sanjiang Plain, Heilongjiang Province, China. The site has an elevation of 55–57 m, with a mean annual precipitation of 565–600 mm and a mean annual temperature ranging from 1.4°C to 4.3°C. The highest temperatures occur in July (21°C–22°C), while the lowest temperatures are recorded in January (−21°C to −18°C). The region experiences a temperate humid to sub‐humid continental monsoon climate, with continuous snow cover from November to April and sub‐zero temperatures persisting from November to May.

We selected five plots within a wetland dominated by *Calamagrostis angustifolia*. Each plot measured 100 m × 150 m, with a minimum separation of 50 m between plots, which supported distinct soil animal communities. We collected both winter‐active and inactive springtails simultaneously from these plots on December 15, 2019, when the average ground temperature was −5.8°C and snow cover ranged from 5 to 15 cm.

### 2.2 Collection of Springtail Species and Phylogenetic Reconstruction

We collected 10 springtail species belonging to four families: *Tomocerus* cf. *jilinensis* (Tomoceridae), *Desoria ruseki* (Isotomidae), *Desoria* sp.1 (Isotomidae), *Desoria* sp.2 (Isotomidae), *Vertagopus* cf. *laricis* (Isotomidae) (all winter‐active species), and *T. nigrus* (Tomoceridae), *Lepidocyrtus felipei* (Entomobryidae), *Homidia* sp. (Entomobryidae), *Friesea* sp. (Hypogastruridae) and *F*. cf. *major* (Hypogastruridae) (all winter‐inactive species). We extracted springtails from 3 L samples collected at 6–8 m intervals within each plot using Tullgren dry extractors. To minimize the impact of Tullgren extraction on springtail‐associated microbiota, we completed the extraction process within 6 h after litter sampling (Zhu et al. 2021). Collected specimens were preserved in anhydrous ethanol at −80°C, and springtails were identified to species level. Individuals from the same species and plot were pooled and treated as a single sample.

**FIGURE 1:**
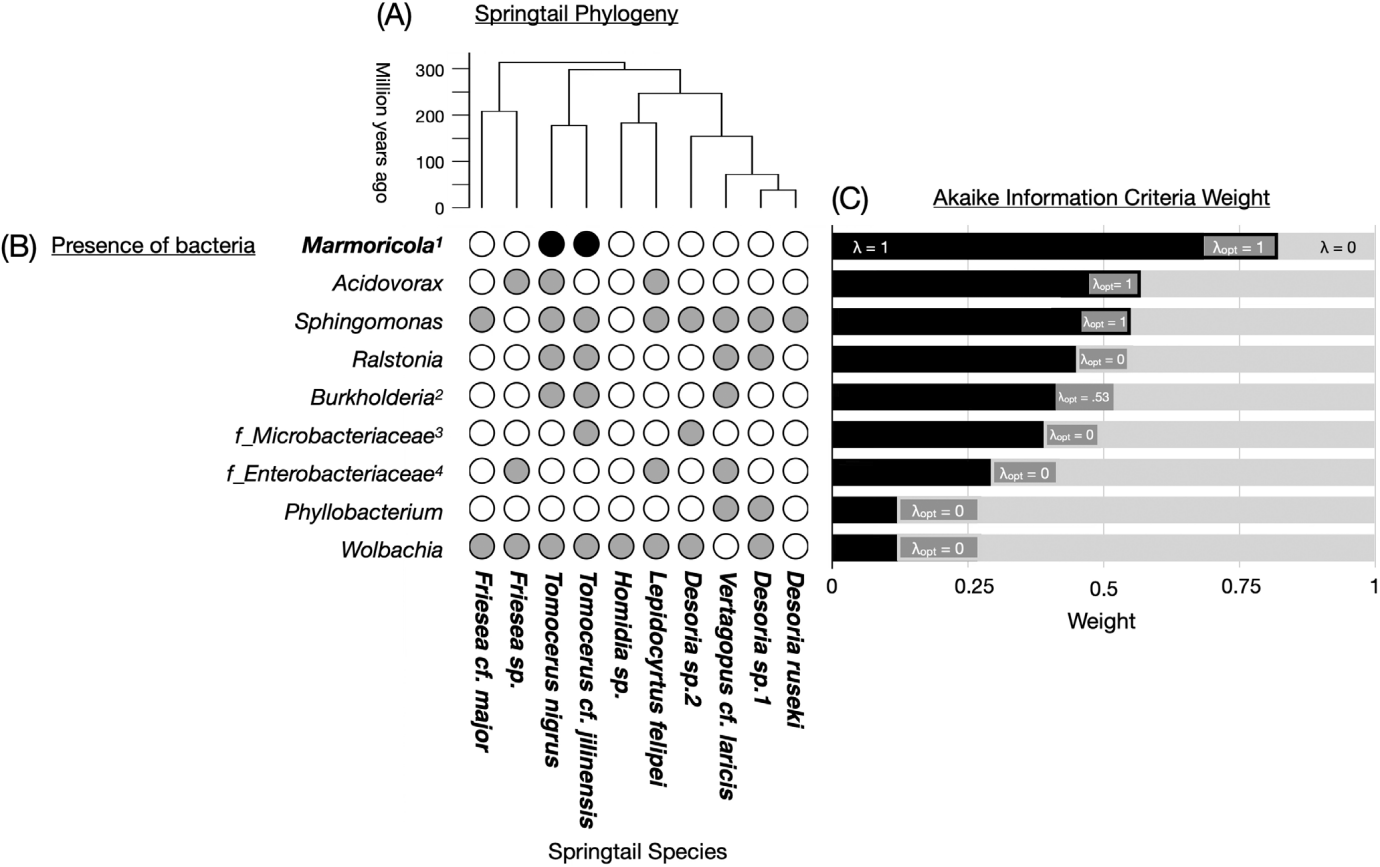
Springtail phylogenetic tree (A) with bacterial genus presence (B) and Akaike information criterion (AIC) weights for phylogenetic signal strength based on Pagel's lambda (λ) (C). Panel (C) compares support for the lambda‐optimized tree (branch lengths adjusted to maximize support for bacterial presence; dark gray), the λ = 1 tree (strong phylogenetic signal; black) and the λ = 0 tree (no phylogenetic signal; light gray). In (A), superscripts indicate: ^1^same for *Mycobacterium*, *Gaiellales*, *Rhodococcus*, and *Vibrionimonas*; ^2^inclusion of *Caballeronia* and *Paraburkholderia*; ^3^unclassified genus within *Microbacteriaceae*; ^4^unclassified genus within *Enterobacteriaceae*. In (B), dot colors indicate presence of bacterial genera with phylogenetic signal (black), no phylogenetic signal (gray), and bacterial absence in springtail species (white).

We reconstructed a phylogenetic tree for the 10 springtail species (Figure 1A) in R 4.0.3. Phylogenetic inference was based on DNA sequences of the 28S rRNA and cytochrome oxidase subunit I (COI) genes for the springtail species of the study region (Supporting Information S1). For springtail individuals analyzed with microbial community metabarcoding in this study, their COI gene was sequenced and then aligned with regional species records. The COI and 28S rRNA alignments were concatenated into a supermatrix for phylogenetic reconstruction using the maximum likelihood method implemented in the *pml* function of the “phangorn” package. We then transformed the resulting phylogram into a chronogram using a strict clock model with four calibrated nodes, applying the *chronos* function from the “ape” package (Paradis et al. 2004). The resulting dated phylogenetic tree (Figure 1A) was used to assess phylogenetic signal of bacterial presence in springtail species. Details on phylogenetic reconstruction are available in the Supplementary material S1.

### 2.3 Microbial Community Associated With Springtails

To eliminate surface microbial DNA contamination, we washed springtail specimens with 0.5% sodium hypochlorite for 15 s, followed by a 1‐min rinse in sterilized water (Anslan et al. 2016; Zheng et al. 2019; Hao et al. 2024). DNA was extracted from pooled specimens of eight adult individuals per species and plot using the Fast DNASpin Kit for Soil (MP Biomedicals, USA). DNA concentrations were measured using a NanoDrop 2000 UV–vis spectrophotometer, and quality was assessed by 1% agarose gel electrophoresis. All procedures were conducted under sterile conditions.

The V3‐V4 region of the bacterial 16S rRNA gene was amplified using the primer pair 338F (5′‐ACTCCTACGGGAGGCAGCAG‐3′) and 806R (5′‐GGACTACHVGGGTWTCTAAT‐3′). PCR amplification followed protocols described in Hao et al. (2024). Positive PCR products were purified using the AxyPrep DNA Gel Extraction Kit (Axygen Biosciences, Union City, CA, USA), quantified using QuantiFluor‐ST (Promega, USA), pooled equimolarly, and subjected to paired‐end sequencing (2 × 300 bp) on an Illumina MiSeq platform (Illumina, San Diego, USA) by Majorbio BioPharm Technology Co. Ltd. (Shanghai, China).

Raw sequencing reads were demultiplexed, quality‐filtered, and merged using Fastp and Flash in Qiime2. The DADA2 pipeline (Callahan et al. 2016) was used to identify chimeric sequences and cluster amplicon sequencing variants (ASVs). Taxonomic assignment was performed using the SILVA 16S rRNA gene database (v.138) with a confidence threshold of 70% (Quast et al. 2013). A total of 2,564,749 high quality sequences were obtained from the 50 springtail samples. For downstream community analyses, ASVs were rarefied to 11,783 sequences per sample. Raw sequence data were deposited in the NCBI SRA database under accession number PRJNA1165424.

### 2.4 Statistical Analysis

All statistical analyses were conducted in R 4.0.3. To assess phylogenetic signal, we used Pagel's lambda (Freckleton et al. 2002; Chen et al. 2017) implemented in the *fitDiscrete* function of the “geiger” package. To conservatively estimate phylogenetic signal for bacterial presence in springtail species, we only considered genera present in all five samples of a given species and detected in at least two species (Figure 1B). We compared the support of the lambda‐optimized tree with the lambda‐1 (indicating phylogenetic signal) and lambda‐0 (no phylogenetic signal) trees using Akaike information criterion corrected for small sample sizes (AICc) and reported the respective weights (Figure 1C).

We quantified bacterial alpha diversity using the Chao 1 and Shannon indices. The Chao 1 index estimates overall species richness by accounting for rare species that may be underestimated during sampling, while the Shannon index incorporates both richness and evenness, making it sensitive to subtle community differences caused by changes in the relative abundance of dominant species (Hugerth and Andersson 2017). Differences in bacterial diversity between springtail species and between winter‐active and inactive taxa were tested using Kruskal‐Wallis tests followed by Dunn post hoc tests. Bacterial alpha diversity and community composition were visualized as bar plots and stacked bar plots, respectively, using the “ggplot2” package. We performed Principal Coordinate Analysis (PCoA) on Bray‐Curtis distances using the “vegan” package, with PERMANOVA (Adonis test; *p* < 0.05) to assess differences in bacterial communities between springtail species and between winter‐active and inactive taxa. To identify differentially abundant bacterial genera between these groups, we applied Linear Discriminant Analysis (LDA) with a threshold logarithmic LDA score of 2.0. We calculated bacterial network properties for taxa with relative abundance > 1% using the “igraph” package, based on Spearman correlation (*r* > 0.6 and *p* < 0.05). Bacterial co‐occurrence networks for winter‐active and inactive springtail taxa were visualized using Gephi (v.0.9).

## 3 Results

### 3.1 Phylogenetic Signal of Bacterial Presence

A total of 3279 ASVs and 494 bacterial genera were detected across the 10 springtail species. Phylogenetic signal was tested for 13 bacterial genera (Figure 1B). The genera *Marmoricola*, *Mycobacterium*, *Gaiellales*, *Rhodococcus*, and *Vibrionimonas* exhibited phylogenetic signal, with an optimized lambda value of 1 and a total weight of 0.82 when summed across both lambda‐optimized and lambda‐1 trees (Figure 1C). These bacterial genera were shared between the two *Tomocerus* species. Although the optimized lambda for the presence of *Acidovorax* and *Sphingomonas* was also equal to 1, the support for the lambda‐1 and lambda‐0 trees was indistinguishable. In contrast, the presence of *Enterobacteriaceae* and *Microbacteriaceae* was scattered across springtail phylogeny, with an optimized lambda value of 0.

### 3.2 Bacterial Alpha Diversity

**FIGURE 2:**
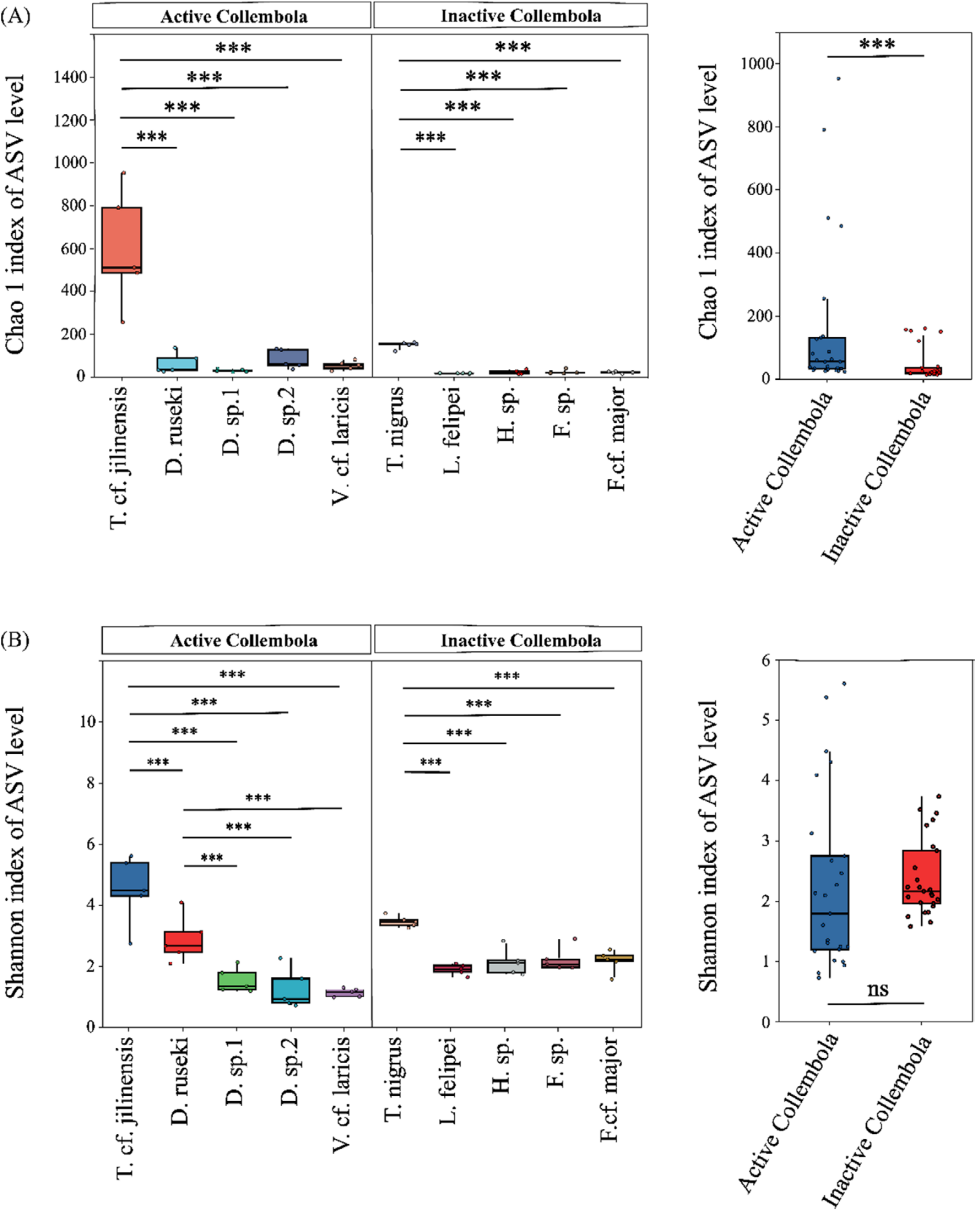
Box plots showing differences in bacterial diversity measured using Chao 1 index (A) and Shannon index (B) across springtail species and between winter‐active and inactive taxa. ***p < 0.001; ns, no significant difference.

Winter‐active springtail taxa exhibited higher bacterial alpha diversity compared to winter‐inactive taxa, as indicated by the Chao 1 index (Kruskal‐Wallis test, *p* < 0.05; Figure 2A), while no significant difference was observed for the Shannon index (Kruskal‐Wallis test, *p* = 0.17; Figure 2B). Among the five winter‐active species, *T.* cf. *jilinensis* displayed the highest bacterial alpha diversity based on both the Chao 1 and Shannon indices (Kruskal‐Wallis test, *p* < 0.05). The Shannon index of *D. ruseki* was *significantly* higher than that of *Desoria* sp.1, *Desoria* sp.2, and *V*. cf. *laricis* (Kruskal‐Wallis test, *p* < 0.05), while no significant difference was detected in the Chao 1 index (Kruskal‐Wallis test, *p* > 0.05). Among winter‐inactive springtail species, *T. nigrus* exhibited higher bacterial alpha diversity compared to *L. felipei*, *Homidia* sp., *Friesea* sp., and *F*. cf. *major* (Chao 1 and Shannon indices; Kruskal‐Wallis test, *p* < 0.05; Figure 2A,B). However, no significant differences in bacterial diversity were detected among *L. felipei*, *Homidia* sp., *Friesea* sp., and *F*. cf. *major* (Kruskal‐Wallis test, *p* > 0.05). Furthermore, 215 ASVs (6.6%) were shared between winter‐active and inactive springtail taxa, whereas 2561 ASVs (78.1%) were exclusively found in winter‐active taxa (Figure S1).

### 3.3 Bacterial Community Composition

**FIGURE 3:**
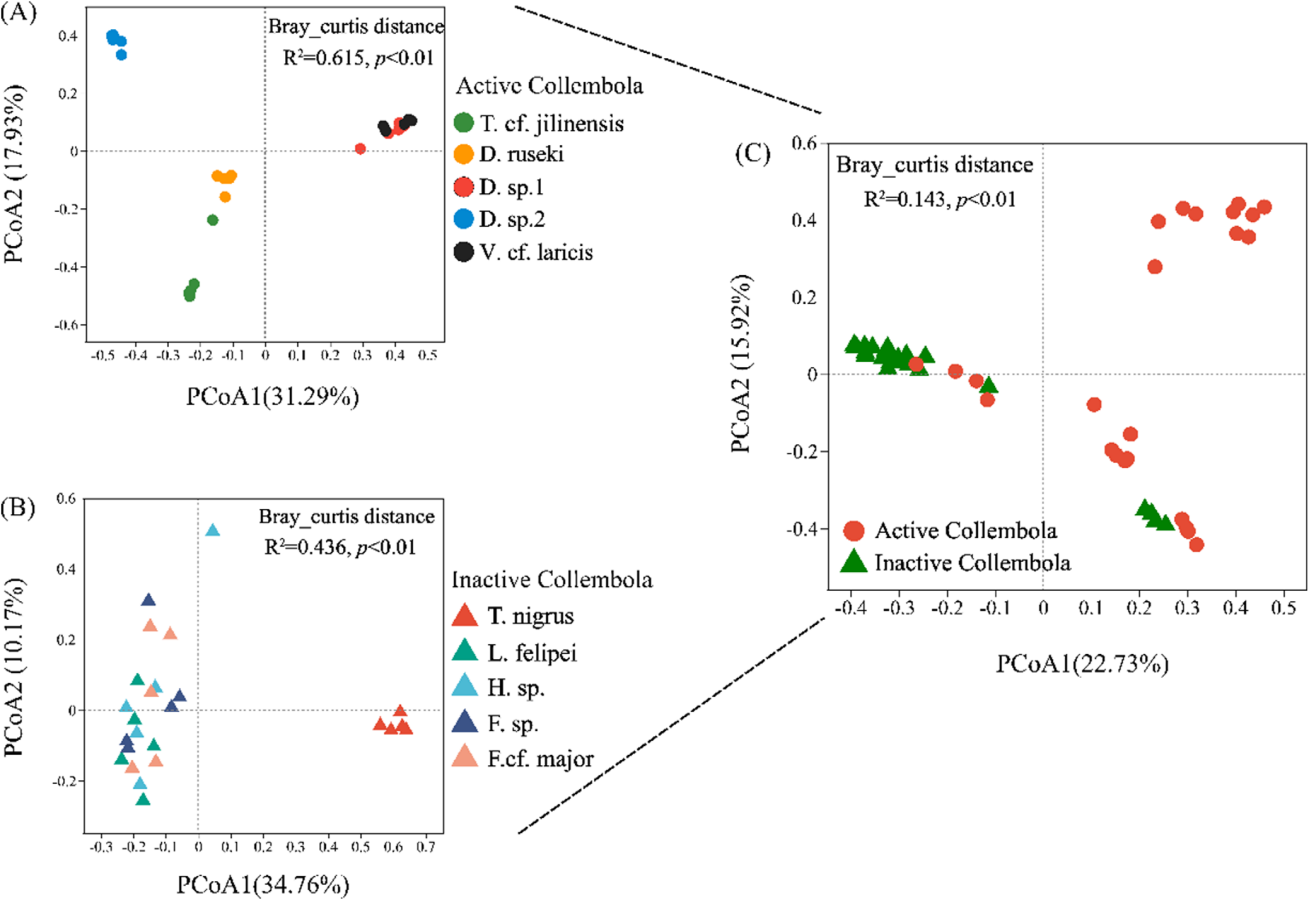
Principal coordinate analysis (PCoA) of bacterial communities associated with springtail species: Winter‐active species (A), inactive species (B), and comparison between active and inactive taxa (C).

**FIGURE 4:**
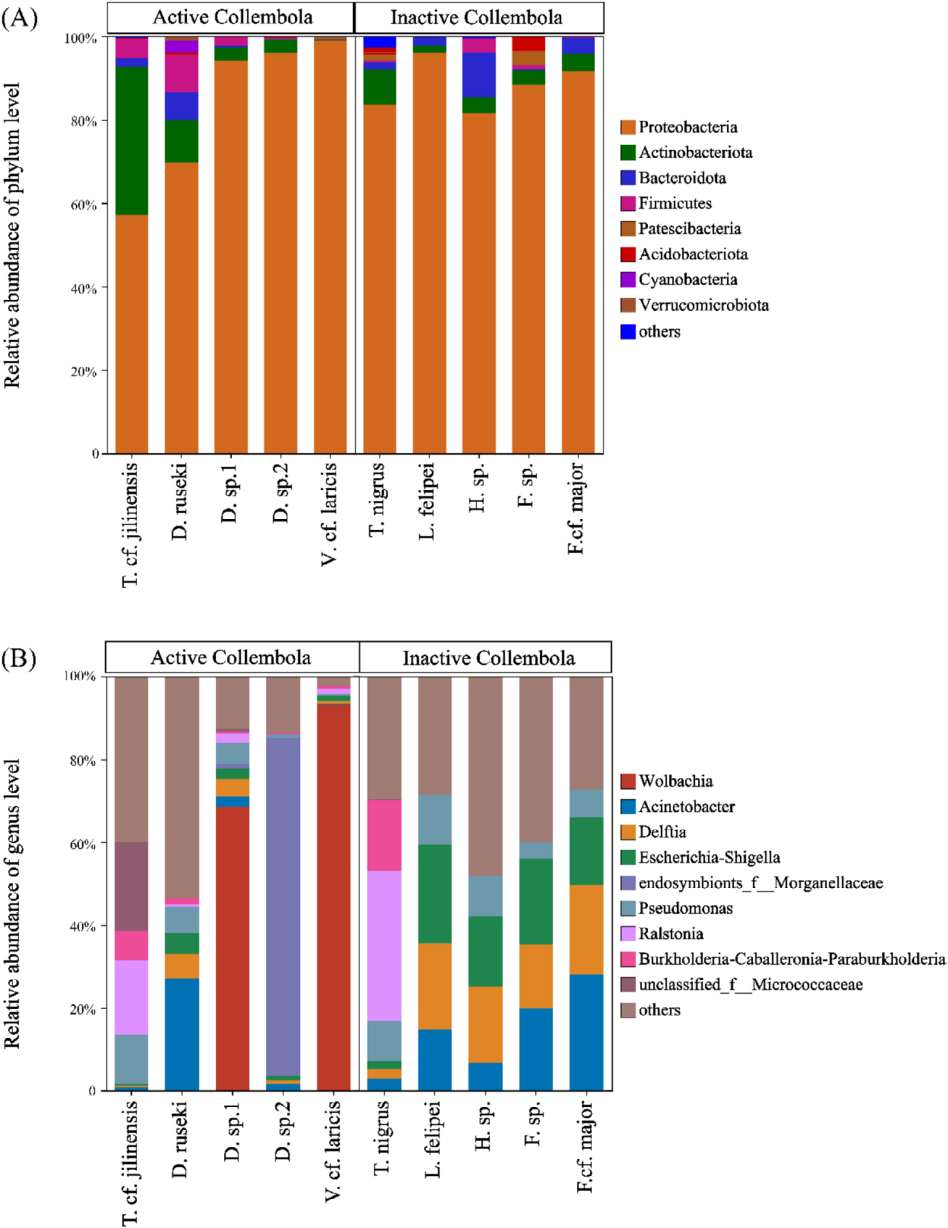
Stacked bar charts showing bacterial community composition at the phylum (A) and genus (B) levels in springtail species. Only phyla with a relative abundance > 1% and genera with a relative abundance > 10% are displayed.

Bacterial communities differed significantly among springtail species in both winter‐active (*R* ^2^ = 0.62, *p* < 0.01) and winter‐inactive taxa (*R* ^2^ = 0.44, *p* < 0.01; Figure 3A,B). Additionally, bacterial communities in winter‐active taxa were significantly different from those in winter‐inactive taxa (*R* ^2^ = 0.14, *p* < 0.01; Figure 3C). The bacterial communities of winter‐active springtails were dominated by the phyla *Proteobacteria* (83.2%) and *Actinobacteriota* (10.5%; Figure 4A), with *Wolbachia* (32.4%) and *Endosymbionts_f_Morganellaceae* (16.5%) as the most abundant genera (Figure 4B). Bacteria communities associated with inactive springtails were primarily composed of *Proteobacteria* (88.21%) and *Bacteroidota* (3.64%; Figure 4A), with dominant genera including *Escherichia‐Shigella* (16.0%), *Delftia* (15.7%) and *Acinetobacter* (14.43%; Figure 4B). Compositional similarity was observed among bacterial genera in the inactive taxa *L. felipei*, *Homidia* sp., *Friesea* sp., and *F*. cf. *major*. However, among winter‐active taxa, only *Desoria* sp.1 and *V*. cf. *laricis* harbored similar bacterial genera (Figure 4B).

A total of 34 bacterial taxa were enriched in winter‐active springtails, whereas 19 taxa were enriched in inactive springtails (Figure S2). Winter‐active taxa were characterized by *Wolbachia*, *endosymbionts_f_Morganellaceae*, and *unclassified_f_Micrococcaceae*, whereas inactive taxa were enriched in *Escherichia‐Shigella*, *Delftia*, *Acinetobacter*, and *Pseudomonas*.

### 3.4 Co‐Occurrence Networks and Predicted Functions of Bacteria

**FIGURE 5:**
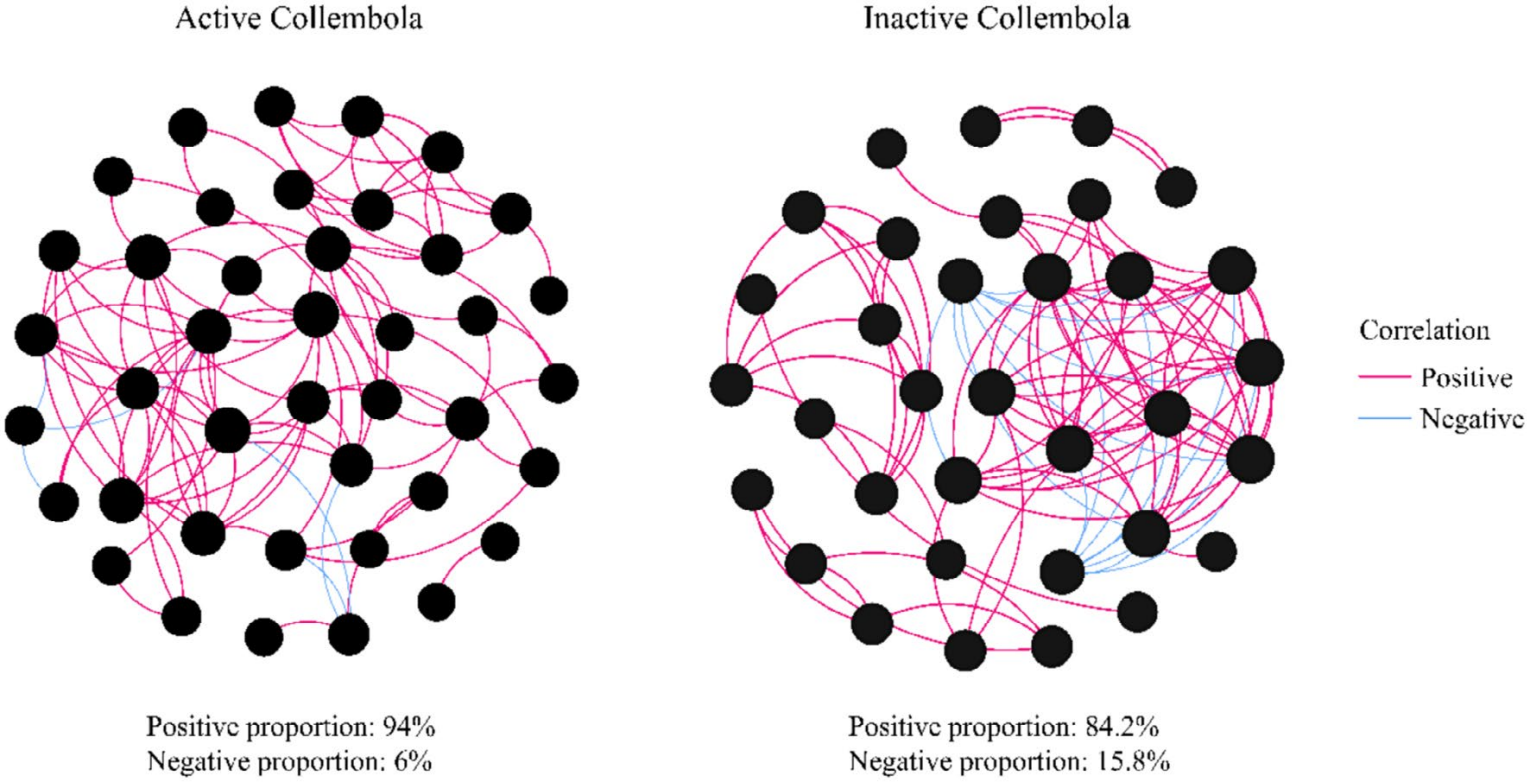
Co‐occurrence networks of bacterial communities associated with winter‐active and inactive springtails. Node size represents the degree of connectivity for bacterial genera with a relative abundance > 1%. Red and blue lines indicate significant positive and negative correlations, respectively.

Bacterial co‐occurrence networks associated with springtails were primarily characterized by positive interactions, which were more frequent in winter‐active taxa compared to inactive taxa (Figure 5). Additional network topology properties are summarized in Table S1.

**FIGURE 6:**
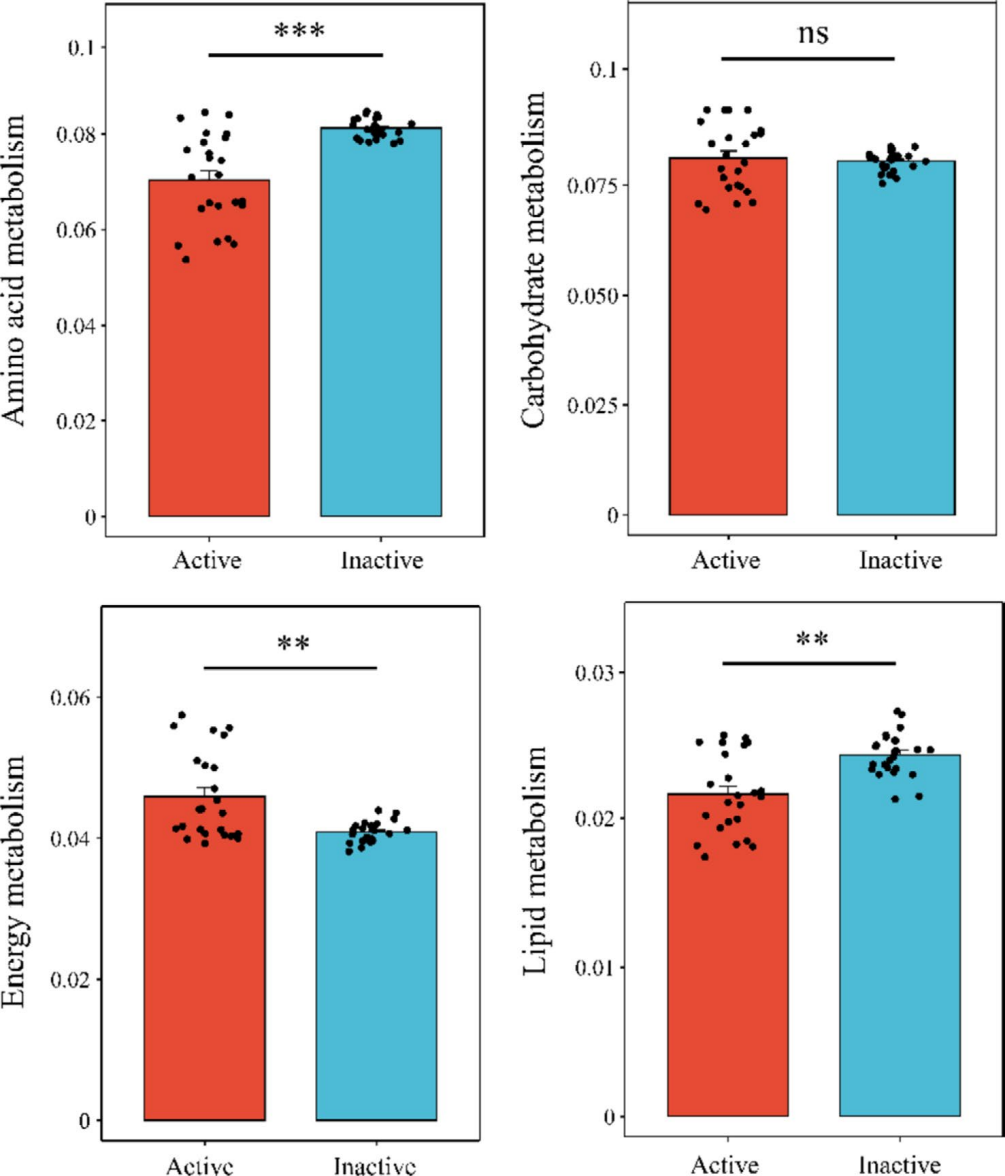
Bar chart illustrating differences in KEGG (level 2) predicted bacterial metabolic functions between winter‐active and inactive springtail taxa. *** *p* < 0.001; ** *p* < 0.01; ns, no significant difference.

Winter‐active springtails exhibited a higher potential for energy metabolism but a lower potential for amino acid and lipid metabolism than inactive springtails (Kruskal‐Wallis test, *p* < 0.05; Figure 6). No significant difference was observed in carbohydrate metabolism between winter‐active and inactive taxa (Kruskal‐Wallis test, *p* = 0.61).

## 4 Discussion

### 4.1 Phylogenetic Signal of Springtail‐Associated Microbiota in the α‐ and β‐Niche Trait Framework

Applying phylogenetic comparative approaches to the α‐ and β‐niche trait framework (Chen et al. 2017; Noske et al. 2024), we aimed to identify microbial taxa that may contribute to springtail winter adaptation. As an initial step, this exploration provides a novel perspective for soil animal research by integrating microbial functional traits with the evolutionary context of microbe‐host interactions. To date, studies on functional traits in springtails and other soil animals have primarily focused on morphological traits such as body length, pigmentation, and optical structure (Xie et al. 2022; Sun et al. 2024). Our study is among the first to assess the phylogenetic signal of microbiota associated with soil animals, highlighting its potential as a trait to elucidate the mechanisms driving belowground animal diversity (Bardgett and van der Putten 2014; Potapov et al. 2021).

Supporting our first hypothesis, the presence of *Marmoricola*, *Mycobacterium*, *Rhodococcus*, and *Vibrionimonas* in springtails exhibited phylogenetic signal. These bacterial genera were generally shared between the two *Tomocerus* species, suggesting evolutionary constraints on the functional roles of these putative symbionts. For example, *Rhodococcus* has been associated with springtail abundance (which may serve as a proxy for host fitness; Liu et al. 2024); however, its actual role remains to be investigated. *Mycobacterium* has also been identified in the guts of springtails and other soil animals, such as earthworms, and is known for its role in cellulose degradation (Pathiraja et al. 2022; Yang et al. 2023). This suggests a potential link between *Mycobacterium* and ecosystem functions, such as litter decomposition. In contrast, while *Burkholderia* has been reported to inhabit the guts of springtails and other insects, playing a role in host nitrogen metabolism (Ohbayashi et al. 2019; Kaltenpoth and Flórez 2020; Stillson et al. 2022), our study found that this genus was not only shared between the two *Tomocerus* species but also with *V*. cf. *laricis*, thereby reducing its phylogenetic signal. The observed patterns of phylogenetic signal suggest that, as β‐niche traits, certain microbial taxa may be functionally important for springtail and may also contribute to broader ecosystem processes. Further physiological experiments and gene expression studies are needed to validate the ecological roles of microbial taxa that are phylogenetically correlated with springtails, providing insights into the functional benefits of animal‐microbial co‐evolution for species adaptation.

According to the α‐ and β‐niche trait framework, phylogenetically labile bacteria function similarly to α‐niche traits, representing potential food resources for springtails. It is well known that microorganisms in the surrounding environment serve as food resources for springtails (Hao et al. 2020). Previous studies have reported that springtails ingest bacteria from the *Enterobacteriaceae* and *Microbacteriaceae* families in their environment (Wüst et al. 2011; Hao et al. 2022). In our study, the presence of these bacteria was scattered across springtail phylogeny. As α‐niche traits, the partitioning of microbial food resources between phylogenetically related springtail species may promote their coexistence, supporting the idea of microbiota as resource traits for soil animals.

### 4.2 Effects of Host Wintering Strategies on Associated Microbiota

Understanding animal‐microbe symbioses is fundamental to studying population maintenance, environmental adaptation, and ecological functioning (Engel and Moran 2013). In this study, we examined the microbiota of springtails exhibiting different wintering strategies (i.e., active vs. inactive). Our findings revealed significant differences in bacterial diversity, community composition, network interactions, and potential metabolism between the two groups, supporting our second hypothesis that animal‐associated microbiota are influenced by host wintering activity. Recent studies have highlighted the importance of microbiota in insect adaptation to cold conditions, particularly in honeybees, bumblebees, and crickets (Ferguson et al. 2018; Mushegian and Tougeron 2019; Bleau et al. 2020; Dittmer and Brucker 2021). In our study, winter‐active springtail species exhibited significantly higher bacterial richness than their inactive counterparts. As generalist feeders, springtails consume a wide range of resources, from litter to microorganisms and even animal prey (Potapov et al. 2021; Hao et al. 2022). The higher microbial diversity in winter‐active species suggests that they may acquire specific microorganisms from their environment to sustain metabolism during harsh winters (Xiang et al. 2019; Hao et al. 2020). Notably, the two *Tomocerus* species, *T*. cf. *jilinensis* and *T. nigrus* , harbored greater bacterial richness than other species. Given their lower trophic position in the soil food web, they may ingest specific microbes from the environment, contributing to this elevated microbial diversity (Potapov et al. 2016; Hao et al. 2020).

Microbial community composition differed between winter‐active and inactive springtails, mirroring findings in house mosquitoes, where diapausing and non‐diapausing individuals displayed distinct gut microbiota (Didion et al. 2021). Consistent with studies of springtail microbiota during the growing season (Zhang et al. 2021; Liu et al. 2024), we found that *Proteobacteria* dominated in winter, suggesting a key role for this phylum in springtail adaptation. However, winter‐active species displayed greater interspecific variation in bacterial composition, whereas the winter‐inactive species *L. felipei*, *Homidia* sp., *Friesea* sp., and *F*. cf. *major* exhibited more uniform microbial profiles, suggesting reduced interactions with their microhabitats. Several bacterial taxa were enriched in winter‐active springtails, including *Wolbachia*, *Morganellaceae*, and *Micrococcaceae*. *Wolbachia*, a widespread endosymbiont in arthropods, can influence host reproductive behavior and alter their life history strategies (Ma et al. 2017; Ju et al. 2020). Interestingly, according to our observations, *D. ruseki*, *Desoria* sp.1, and *V*. cf. *laricis* exhibited high population densities in winter but were absent in summer, suggesting a possible link between *Wolbachia* infection and seasonal population dynamics. Meanwhile, *Micrococcaceae* are known to be involved in nutrient absorption and growth in arthropods (Engel and Moran 2013; Hao et al. 2022). Their enrichment in winter‐active springtails suggests a role in enhancing energy utilization, potentially contributing to host survival under cold conditions.

Network analysis revealed a higher proportion of positive microbial interactions in winter‐active springtails compared to winter‐inactive taxa. This suggests that cooperative interactions between bacteria may facilitate resource utilization and stabilize microbial networks under harsh winter conditions (Castrillo et al. 2001; Morgan‐Richards et al. 2023; Hao et al. 2024). In contrast, winter‐inactive springtail species exhibited a greater number of negative microbial interactions, possibly reflecting compromised microbiota with increased susceptibility to pathogen infection. Potential metabolic functions based on 16S rRNA gene amplicons further supported the role of microbiota in host winter adaptation (Langille et al. 2013). Winter‐active species harbored a higher relative abundance of bacteria associated with energy metabolism but showed lower representation of lipid metabolism pathways compared to winter‐inactive species. This suggests that winter‐active springtails rely on microbial contributions to meet their energy demands, whereas winter‐inactive species maintain a low metabolic rate, possibly relying on lipid reserves for energy conservation. Further experimental studies are needed to confirm the specific metabolic roles of microbes in nutrient acquisition and energy regulation in winter‐adapted arthropods.

## 5 Conclusions

In this study, we explored the relationships between host phylogeny, winter activity, and associated microbiota in springtails, an example of soil animals adapted to cold environments. Our findings revealed that certain bacterial taxa exhibited phylogenetic signal in their association with springtail species, suggesting that these microbes may play functional roles in host adaptation, as framed within the α‐ and β‐niche trait concept. Springtail species using different wintering strategies showed distinct bacterial diversity, community composition, network interactions, and metabolic potential. Building on our previous seasonal research (Hao et al. 2024), these findings underscore the potential role of microbiota in enhancing the ecological success and adaptation of soil animals to extreme conditions. The observed differences in microbiota between winter‐active and inactive springtails suggest that microbiota not only support host activity at low temperatures but also contribute to general soil biodiversity (Liu et al. 2024) and ecosystem processes. To further elucidate the functional roles of microbiota, future research should integrate experimental measurements of physiological traits, such as metabolic rates, lipid reserves, and gene expressions, to validate their contributions to host metabolism and winter adaptation.

## Author Contributions

**Cao Hao:** conceptualization (lead), data curation (lead), formal analysis (equal), investigation (lead), software (equal), validation (equal), visualization (lead), writing – original draft (lead), writing – review and editing (equal). **Bing Zhang:** methodology (supporting), software (equal), writing – review and editing (equal). **Pingting Guan:** methodology (supporting), software (equal), writing – review and editing (equal). **Zhijing Xie:** methodology (supporting), software (equal), writing – review and editing (equal). **Guoliang Xu:** methodology (supporting), software (equal), writing – review and editing (equal). **Donghui Wu:** conceptualization (supporting), funding acquisition (lead), investigation (supporting), methodology (supporting), project administration (lead), resources (lead), software (equal), supervision (equal), visualization (supporting), writing – original draft (supporting), writing – review and editing (equal). **Ting‐Wen Chen:** conceptualization (lead), data curation (supporting), formal analysis (equal), methodology (lead), software (equal), supervision (equal), validation (equal), visualization (lead), writing – original draft (lead), writing – review and editing (equal).

## Conflicts of Interest

The authors declare no conflicts of interest.

## Supporting information


Data S1:

## Data Availability

Raw sequence data are deposited in the NCBI SRA database under accession number PRJNA1165424. Other data are provided as the online Supporting Information S1.
