# Supplementary material for: Microbiota as Potential Functional Traits Facilitating Springtail Activity in Winter
Source: Ecol Evol. 2025 May 19;15(5):e71448. doi: 10.1002/ece3.71448 (PMC12086981; doi:10.1002/ece3.71448)
Supplement: Supplementary file 1 — Data S1: [file ECE3-15-e71448-s001.zip › Supplementary_material_20241203_TC.docx]

**Supplementary material**


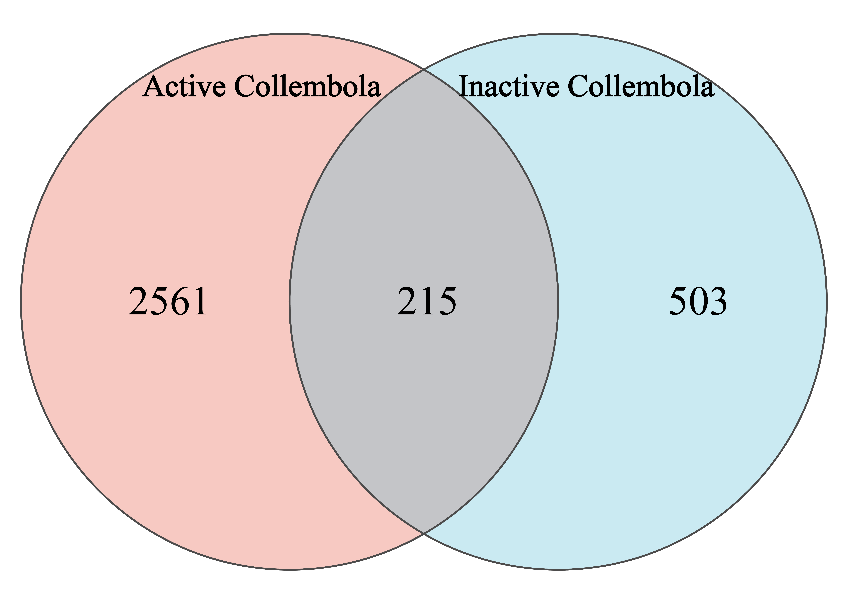


**Figure S1** Venn diagrams showing the overlap and unique bacterial ASVs between winter-active and inactive springtails.


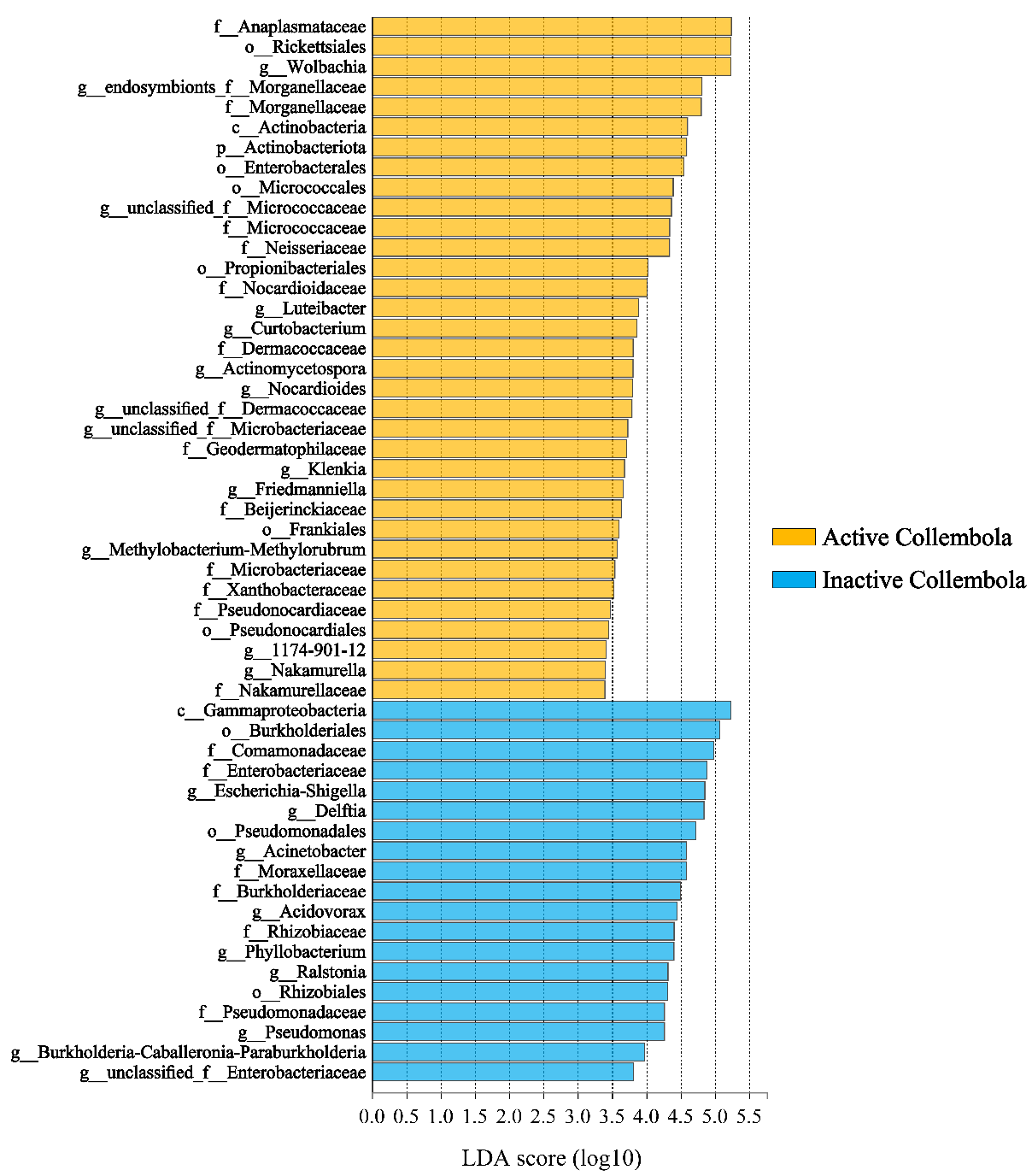


**Figure S2** Linear discriminant analysis (LDA) plot showing indicator bacterial taxa distinguishing winter-active and inactive springtail taxa. The discrimination threshold was set at 2.0 as the logarithm of the LDA score, which indicates the degree of representation of bacterial taxa for treatments.

**Table S1** Properties of bacterial network topologies in winter-active and inactive springtail taxa.

| **Property** | **Active springtail network** | **Inactive springtail network** |
| --- | --- | --- |
| Modularity | 0.56 | 0.6 |
| Nodes | 41 | 34 |
| Edges | 100 | 101 |
| Degree | 4.9 | 5.9 |
| Path length | 2.5 | 1.5 |
| Diameter | 6.0 | 4.0 |
| Positive edges | 94% | 84.2% |
| Negative edges | 6% | 15.8% |
